# Evaluating the Effect of National Background Check Program on Nursing Home Deficiency Citations

**DOI:** 10.1111/1475-6773.70108

**Published:** 2026-04

**Authors:** Junjie Gai, Hari Sharma, Brian Kaskie, Gerald John Jogerst

**Affiliations:** 1 Department of Health Management and Policy, College of Public Health, The University of Iowa, Iowa City, Iowa, USA; 2 Department of Family Medicine and Psychiatry, University of Iowa Carver College of Medicine, Iowa City, Iowa, USA

**Keywords:** abuse and neglect, background checks, nursing homes, quality of care, regulatory impact

## Abstract

**Objective:**

To evaluate the impact of the National Background Check Program (NBCP) on nursing home (NH) health deficiencies and citations related to abuse, neglect, and exploitation.

**Study Setting and Design:**

This study uses the Callaway and Sant'Anna Difference-in-Differences (CSDID) quasi-experimental method to analyze data from US nursing homes from 2009 to 2016. The study includes nursing homes from 18 states that received NBCP grants as treatment group and nursing homes from 24 states that did not receive NBCP grants as control group. We exclude eight pilot NBCP states.

**Data Sources and Analytic Sample:**

We used facility-level deficiency data from NH Care Compare (CC), NH characteristics data from Certification and Survey Provider Enhanced Reports (CASPER), and Alzheimer's Disease and Related Dementias diagnosis data from Minimum Data Set (MDS) assessments, covering 96,261 nursing home-year observations.

**Principal Findings:**

Overall, NBCP implementation was associated with a significant reduction in health deficiencies (−0.760, *p* < 0.01) and a decrease in the probability and number of citations for abuse, neglect, and exploitation (−0.029, *p* < 0.01; −0.048, *p* < 0.01). Subgroup analyses showed that NBCP was associated with reductions in health deficiencies in nursing homes, regard-less of whether they had a high or low census of residents with Alzheimer's Disease and Related Dementias, and in both metropolitan and nonmetropolitan areas. However, the effects varied across states depending on when they adopted NBCP.

**Conclusions:**

Our findings suggest that NBCP is an effective regulatory tool for improving nursing home deficiencies and reducing incidents of abuse-related violations. We need more research to assess if background check programs improve nursing home quality using resident-level outcomes.

## 1 ∣ Introduction

Over a million individuals reside in nursing homes (NH) across the United States [1], and incidents of assault, deaths due to mistreatment and neglect, and financial exploitation frequently make headlines in newspapers [2-4]. In the United States, over 20% of NH are cited annually for abuse, neglect, and exploitation [5]. Residents in nursing homes are vulnerable to various forms of abuse, including physical harm that causes pain or injury, neglect that leads to inadequate provision of necessary services, psychological abuse that results in stress and depression, and financial exploitation that can deplete their life savings [6]. Numerous studies have shown that abuse and neglect of older adults are associated with higher rates of mortality, fractures, depression, pressure ulcers, dehydration, malnutrition, and lower quality of life [6-13].

Older age, combined with declines in physical and cognitive abilities, makes nursing home residents highly vulnerable to abuse and neglect [12]. Risk of abuse and neglect is even greater for individuals with Alzheimer's Disease related dementias (ADRD), as they are unlikely to recognize and report such incidents [8, 14-17]. More than 50% of NH residents are affected by ADRD [18, 19].

Previous research has associated abuse and neglect with staffing and a recent Office of Inspector General (OIG) report found that many employees convicted of abuse had prior criminal convictions that could have been identified through thorough background checks [11, 20]. Strengthening background checks to ensure that employees with direct access to NH residents do not have a history of criminal activity or abuse could be a critical first step in reducing the prevalence of abuse and neglect in NH. Moreover, employing staff without a history of criminal or abusive behavior may also improve patient outcomes such as pressure sores [21].

The National Background Check Program (NBCP) is a voluntary program that aims to enhance the safety and quality of care in long-term care (LTC) facilities across the United States [22]. NBCP was piloted in 2005 in seven states (Illinois, Alaska, New Mexico, Nevada, Michigan, Wisconsin, and Idaho). The Centers for Medicare and Medicaid Services (CMS) opened NBCP in 2010 to all states under the Affordable Care Act (ACA) [22]. NBCP aims to protect vulnerable residents by ensuring all prospective NH employees with direct patient access undergo comprehensive background checks before hiring. These checks include fingerprinting, criminal history record checks, and cross-referencing with abuse and neglect registries. By preventing individuals with histories of abuse, neglect, exploitation, or other severe offenses from entering the LTC workforce, the NBCP seeks to improve care standards and avoid harm to residents. Since its inception, the NBCP has been implemented in 27 states and the District of Columbia (DC), supported by three-year federal grants to develop the necessary infrastructure and systems for effective background screening. This initiative is part of a broader effort by CMS to strengthen regulatory oversight and improve the quality of care in nursing homes and other LTC settings [22, 23].

The NBCP operates as a workforce-screening and regulatory mechanism designed to prevent individuals with disqualifying crimes or abuse histories from gaining direct access to residents. NHs were required to not hire or employ individuals with adverse actions even prior to the NBCP. If NHs employed individuals with adverse actions, they could be issued a deficiency citation (F225 tag). However, practices varied widely across different states in scope, depth, and enforcement [5]. The NBCP strengthens these existing requirements by expanding the depth and scope of background screening, integrating multiple state and federal data systems, and enabling continuous monitoring (“rap-back”) for new criminal offenses. By improving hiring safeguards and increasing the likelihood that unqualified workers are screened out, the NBCP creates a regulatory pathway to reducing health deficiency citations and citations related to abuse, neglect, and exploitation. Our study evaluates how this regulatory mechanism translates into measurable outcomes in health and abuse related deficiencies.

An initial evaluation of the NBCP conducted by the OIG found that approximately 80,000 applicants were ineligible to serve in LTC settings in eight states based on the background checks [23]. A recent study that evaluated the effects of NBCP on care quality in NH using traditional two-way fixed effects (TWFE) regressions found that NBCP participation was associated with fewer deficiencies and higher star ratings [24]. However, the heterogeneity of effects across time and groups is unclear. In addition, we do not have evidence on the effects of NBCP on abuse, neglect, and exploitation—the key aspects of care targeted by NBCP. Our study provides a comprehensive understanding of NBCP effectiveness on aspects of care likely to be affected by NBCP by evaluating the impact of the NBCP on health deficiencies, deficiencies related to abuse, neglect and exploitation, overall and across several subgroups by ADRD census, and metropolitan and non-metropolitan areas.

Each year, state survey agencies conduct NH health inspections and may inspect NH more often if the NH is performing poorly or if there are complaints or facility-reported incidents. If an inspection team finds that a NH does not meet a specific federal standard, it issues a citation for the health deficiency. The federal or state governments may impose penalties on NH for serious citations or for citations that the NH has not corrected for a long time [25]. Using the annual NH inspection surveys and latest quasi-experimental difference-in-differences (DID) designs that account for variation in treatment timing and treatment effect heterogeneity [26], we assess the effects of the NBCP on NH health deficiency citations and citations for abuse, neglect and exploitation, overall, and across several subgroups.

Our study provides several novel contributions using advanced research designs and validates our findings using a series of robustness checks. First, we employ the latest DID designs to address the bias in traditional TWFE models when treatment effects are heterogeneous across states and time. This approach allows us to reliably estimate the average treatment effect for each adoption cohort and aggregate these effects. Second, we look at broader outcomes (all health deficiencies) as well as those that are likely to be affected by NBCP (citations related to abuse, neglect, and exploitation) to assess the extent to which NBCP is working to improve resident welfare. Third, we assess differential effects not only across time and groups but also evaluate NBCP effects for different subgroups by ADRD census and metropolitan status. These subgroup analyses provide additional insight for policymakers to evaluate the extent to which NBCP impacts vary across time and groups.

## 2 ∣ Methods

### 2.1 ∣ Data and Variables

#### 2.1.1 ∣ Outcome Variables

To identify citations related to health deficiencies, abuse, neglect, and exploitation, we utilize citation information from Nursing Home Care Compare (CC), which tracks citations using F-tags to represent instances of abuse, neglect, and exploitation. For health deficiencies, we use all F-tags cited. Using the literature, we identify citations for abuse, neglect, and exploitation using the F-tags F221, F222, F223, F224, F225, and F226 [19, 27, 28]. We provide the detailed description of these tags in the Table S1. In 2017, CMS introduced new F-tags, with codes ranging from F600 to F610, replacing the older system [28, 29]. Since NH inspectors started using new F-tags in late 2017, our primary analysis focuses on the period between 2009 and 2016, when the old F-tags were used. Although old and new F-tags are meant to capture similar issues, there are some differences between old and new F-tags in terms of their definitions. Using these citation tags, we created three separate outcome measures: (a) the total number of health deficiency citations for NH (a continuous measure); (b) citations for abuse, neglect, and exploitation (a binary variable equal to 1 if cited, 0 otherwise), and the number of citations for abuse, neglect, and exploitation (a continuous measure). Our analysis uses both standard and complaint surveys and includes any health deficiencies issued to a facility.

#### 2.1.2 ∣ Control Variables

We obtain information on payer mix (Medicare, Medicaid, and Private), number of beds, ownership, occupancy rate, and staffing hours per resident day (HPRD) for Registered Nurses (RNs), Licensed Practical Nurses (LPNs), and Certified Nursing Assistants (CNAs) from the Certification and Survey Provider Enhanced Reports (CASPER) data. Consistent with the literature, we calculate HPRD for each full time equivalent (FTE) staff type as: total FTE*70/Total residents*14 [30].

We identify facilities with high or low ADRD census using NH resident level MDS from 2010 to 2016. Based on the literature, we identified ADRD using the ICD 9/10 codes for Alzheimer's disease (ICD-9: 331.0; ICD-10: G30.0, G30.1, G30.8, G30.9), and related dementias including vascular dementia (ICD-9: 290.40, 290.41; ICD-10: F01.50, F01.51), Lewy body dementia (ICD-9: 331.82; ICD-10: G31.83), frontotemporal dementia (ICD-9: 331.19; ICD-10: G31.09), and primary progressive aphasia (ICD-9: 331.11; ICD-10: G31.01) and active diagnosis of ADRD in MDS [17, 31]. We calculate the low/high ADRD census by the median percentage of the ADRD resident population in nursing homes for each year. We define competition using the Herfindahl–Hirschman Index. We obtain the local area unemployment rate at county-level data from the U.S. Bureau of Labor Statistics and publicly available crime data per 100,000 people in each state from the Federal Bureau of Investigation [32, 33]. Using the 2013 Rural–Urban Continuum Codes (RUCC), we identify nursing homes in metropolitan and nonmetropolitan areas for subgroup analysis [34, 35]. We link the CC, CASPER, and MDS datasets using the federal nursing home provider number.

### 2.2 ∣ Sample

#### 2.2.1 ∣ Treatment Group

We include 18 states that implemented the NBCP program for the first time between 2010 and 2015 in the treatment group (Delaware, Connecticut, Florida, Missouri, Rhode Island, California, Oklahoma, Kentucky, Utah, North Carolina, Maine, West Virginia, Georgia, Minnesota, Hawaii, Ohio, Oregon, and Kansas).

#### 2.2.2 ∣ Control Group

States in the control group consist of 24 states that did not participate in the NBCP program (Alabama, Arizona, Arkansas, Colorado, Indiana, Iowa, Louisiana, Massachusetts, Montana, Maryland, Nebraska, New Hampshire, New Jersey, New York, North Dakota, Pennsylvania, South Carolina, South Dakota, Tennessee, Texas, Virginia, Washington, and Wyoming).

We exclude NBCP pilot states (Alaska, Illinois, New Mexico, Michigan, Nevada, Wisconsin, and Idaho) from our main analysis since they were already exposed to the treatment prior to 2010 [36]. Finally, we do not include Idaho and Mississippi since they are still working on the buildup of the programs and extended their grant periods to 2024 [36]. Our study includes 96,261 observations at the NH level from 2009 to 2016.

### 2.3 ∣ Statistical Analyses

Since the NBCP grants were awarded in 2010, 2011, 2012, 2013, and 2015, our analysis evaluates NBCP's causal impact on the incidence of citations for health deficiencies, abuse, neglect, and exploitation within NHs using the Callaway and Sant'Anna DID (CSDID) methodology [26]. CSDID is a robust statistical technique designed to estimate causal relationships by comparing changes over time between treatment groups (those affected by the policy or intervention) and control groups (those not affected). This approach is particularly suited to our study as it allows for evaluating policy impacts over multiple treatment groups and time periods, accommodating the staggered implementation of the NBCP grants. CSDID improves on the generalized DID with TWFE designs that are problematic when treatment effects are heterogeneous across groups or time. Using CSDID, we aim to isolate the effect of NBCP, providing a more accurate assessment of its impact on NH citations. In our analysis, NH in states that implemented NBCP were designated as treated, while NH in states that did not adopt NBCP (never treated) served as our primary controls (Model 1). We also generate event study plots from the CSDID model by aggregating group time average treatment effect on the treated (ATT) to examine the dynamic trends of the effect of NBCP on citations. We also estimate heterogeneity in treatment effects by grouping states into different groups based on the year of NBCP adoption. All analyses cluster standard errors at the state level.

We conduct a series of robustness checks using different specifications or samples. First, to support the assumption of parallel trends, we use two distinct approaches. Using the traditional approach, we review the event study plots for any potential trends. We also use a recently developed approach to evaluating the extent to which potential violations of the parallel trends assumption affect our findings using the Honest Difference-in-Differences (honestdid) approach [37]. Use of HonestDID allows us to move away from simply testing whether any years in the baseline period are different from zero to assessing the extent to which violations of parallel trends affect our inferences. This approach to assessing parallel trends is more appropriate in our situation where we have a large number of years and sample size. Second, we estimate group ATT based on the NBCP states using both weighted and unweighted averages. Third, we add a limited set of covariates (time-invariant facility-level covariates) and the full set of controls described in the data and variables section. We use a doubly robust DID approach while controlling for covariates. Although the addition of covariates is not required in difference-in-differences regressions, we can assess the robustness of our findings conditioning on pre-NBCP covariates. Fourth, we include both never-treated and not-yet-treated states in the control group. Our preferred specification for the control group includes states that never enacted NBCP policies, but we evaluate if including states that enacted NBCP later along with those that never enacted NBCP as controls changes our findings. Fifth, we perform subgroup analyses by ADRD census and metropolitan status. We evaluate NBCP effect on low/high ADRD census in nursing homes, as identified in the MDS. Evidence indicates that ADRD residents in NH are more vulnerable to abuse, neglect, and exploitation due to cognitive impairments that limit their ability to report mistreatment [7, 13-16]. Therefore, we expect greater improvement in health deficiency citations for NH with high ADRD census because of more strict background checks. We also assess the heterogeneity in effect by categorizing facilities as metropolitan (RUCC: 1 to 3) and nonmetropolitan (RUCC: 4 to 9) areas. NH in nonmetropolitan areas face challenges in providing quality care due to staffing shortages [38, 39]. Therefore, we expect a difference in effect in metropolitan and nonmetropolitan areas. Sixth, we drop F221 and F222 tags which may capture inappropriate care practices rather than abuse or neglect in our evaluation of citations related to abuse and neglect [40]. Seventh, following the literature [41], we assess the deficiency score associated with the citations used in our main analysis to assess whether NBCP also reduces the severity of regulatory findings. Eighth, although they are a small proportion of the overall sample, we exclude hospital-based NH because these facilities may differ from freestanding NH in staffing structure, patient acuity, and regulatory oversight [40]. Ninth, we use data from 2011 to 2016 to avoid potential confounding from major CMS regulatory changes related to the five-star rating in 2009 as well as resident assessments in 2010 [42]. Finally, we conduct a placebo test using environmental citations. Environmental citations refer to deficiencies in environmental safety in NH that are unlikely to be directly affected by staff background checks [43]. If we find that NBCP has no impact on environmental citations, it may further validate our causal interpretation of NBCP on health deficiency citations.

All analyses are performed using Stata 18.

## 3 ∣ Results

### 3.1 ∣ Sample Description

Table 1 summarizes the baseline descriptive statistics of outcomes and characteristics of NH in NBCP and non-NBCP states in 2009. NHs in NBCP states had a slightly higher average number of health deficiencies (8.48) compared to non-NBCP states (6.97). The probability of receiving citations for abuse, neglect, and exploitation was 0.29 in NBCP states and 0.25 in non-NBCP states. Facilities in NBCP states had fewer beds on average (99.46) compared to those in non-NBCP states (115.32). Additionally, NBCP states had a higher proportion of for-profit nursing homes (71.46%) and a lower percentage of government-run facilities (5.37%) than non-NBCP states (65.84% and 6.82%, respectively). NHs in NBCP states had slightly higher CNA HPRD (2.39) than non-NBCP states (2.22), although RN and LPN HPRD were relatively similar across both groups. NHs in NBCP states had higher average county-level unemployment rates (9.92%) and slightly lower state-level crime rates per 100,000 population (414.06) compared to non-NBCP states (8.21% and 412.93, respectively). Finally, NBCP states had a slightly higher proportion of facilities in metropolitan areas (73.25% vs. 70.11%) versus nonmetropolitan areas.

### 3.2 ∣ Callaway and Sant'Anna DID Estimates

Table 2 shows the estimated effects of the NBCP on the number of health deficiencies, the number and probability of citations for abuse, neglect, and exploitation. Model 1 represents CSDID analysis without covariates. Model 2 includes adjustments for number of beds, profit status, HHI index, chain membership, continuing care retirement community status, having special care units, and acuity scores. Model 3 includes two groups of control groups: never-treated and not-yet-treated NH. NBCP implementation is associated with a 0.76 reduction (a 10% decline relative to sample mean of 7.66) in the number of health deficiencies in Model 1 (*p* < 0.01) and the findings are largely similar when controlling for select covariates (Model 2) and when using both never treated and not-yet-treated NH as controls (Model 3). The NBCP was also associated with a 2.9 percentage point reduction in the probability of NH receiving citations for abuse, neglect, and exploitation (*p* < 0.01). Finally, the number of citations for abuse, neglect, and exploitation also decreased by 0.048 (a 14% decline relative to sample average of 0.35) following NBCP implementation in Model 1 (*p* < 0.01) and these results are similar across Model 2 and 3.

**FIGURE 1 ∣:**
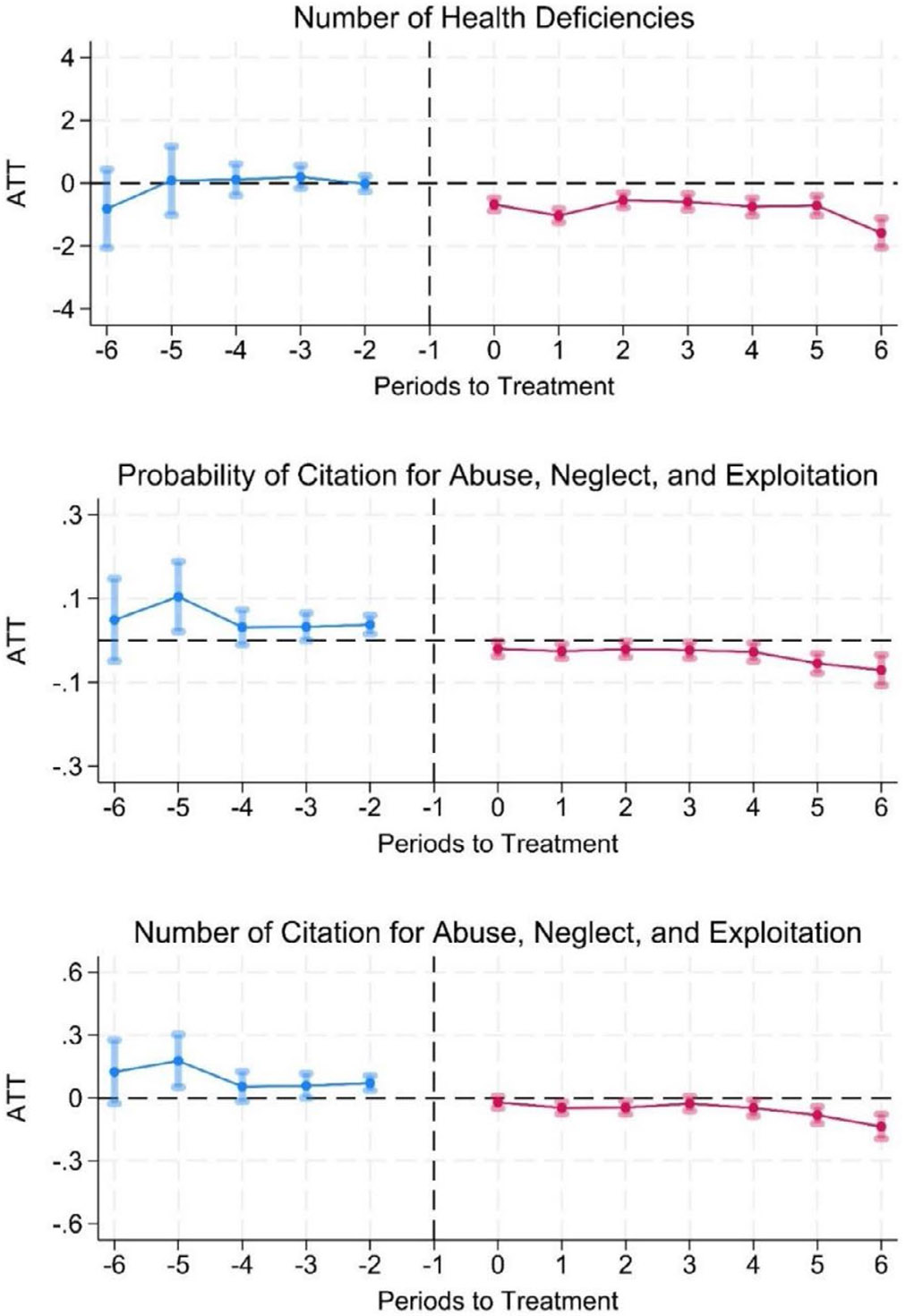
Event study plots of the effects of NBCP on health-related deficiency citations. *Note:* Event study estimates and 95% confidence intervals of the number of health deficiencies, probability and number of citations for abuse, neglect, and exploitation. The reference period is the year before NBCP implementation (Period-1), and the treatment period marks the year of implementation (Period-0). The plots illustrate the average treatment effect on the treated (ATT) over multiple periods before and after the intervention, focusing on three outcomes: the number of health deficiencies, the probability of citation for abuse, neglect, and exploitation, and the number of such citations. Among the three outcomes, the ATT estimates are plotted with 95% confidence intervals, showing temporal dynamics and indicating whether NBCP implementation resulted in significant improvements in nursing home quality.

Figure 1 shows the event study plot of NBCP implementation effects on nursing home citations along with 95% confidence intervals. The reference period is the year prior to NBCP implementation (*t* – 1), and the treatment period starts the year of implementation (Period 0). The plots illustrate the ATT over years before and after the intervention, focusing on three outcomes: the number of citations for health deficiencies, the number and probability of citation for abuse, neglect, and exploitation. In all plots, we observe no differences in outcomes in the years prior to NBCP implementation but we observe meaningful differences in outcomes following the NBCP implementation across years.

We present the findings from several subgroup analyses in Table 3. We find that NBCP significantly reduced the number of health deficiencies by 0.812 for NH with high ADRD census (*p* < 0.01) and by 0.645 for NHs with low ADRD census (*p* < 0.01). The probability and number of citations for abuse, neglect, and exploitation also decreased significantly across both subgroups. Similarly, NHs in metropolitan areas experienced a 0.78 reduction in health deficiencies (*p* < 0.01), and NHs in nonmetropolitan areas experienced a 0.696 reduction in health deficiencies (*p* < 0.01). In both metropolitan areas and nonmetropolitan areas, we observe significant reductions in the probability and the number of citations for abuse, neglect, and exploitation.

The findings from our robustness checks are shown in a series of Supplementary Tables and Figures. The results from honestDID suggest that we can allow minor deviations from parallel trends assumption and still be confident that our findings are valid (Figure S1). The honestDID results show that our estimated treatment effects remain statistically significant even when we allow post-treatment deviations from parallel trends to be larger than the largest difference observed in the pre-treatment coefficients for the health deficiencies, and a little over half of the largest difference observed in pre-treatment coefficients for abuse/neglect results. Table S2 shows a considerable heterogeneity in treatment effects across the states that adopted NBCP over different years, with significant reductions in health deficiencies as well as probability of citations for groups adopting NBCP in 2010, 2011, and 2012. However, we did not see significant impact of NBCP on health deficiencies for Ohio and Oregon that adopted NBCP in 2013 and Kansas that adopted NBCP in 2015. Similarly, we observe heterogeneity in effect of NBCP when we controlled for the full set of covariates (Table S3). Table S4 shows that the NBCP's effects remain similar to our main results when controlling facility-level covariates. Table S5 shows that the estimated effects of the NBCP are consistent with main findings when excluding F221 and F222. We also find that NBCP reduces total deficiency scores as well as abuse-related deficiency scores (Table S6). Table S7 shows that the effects of the NBCP remain consistent with main findings when hospital-affiliated NHs are excluded from our models. The findings in Table S8 are broadly similar to our main findings when we limit the study period from 2011 to 2016. Finally, as expected, our placebo test suggests that NBCP has no impact on environmental citations (Table S9).

## 4 ∣ Discussion

Our analysis showed that NHs in NBCP states experienced 0.76 fewer health deficiencies, on average, compared to those in non-NBCP states. The event study plots show a consistent decline in the number of health deficiencies including those related to abuse, neglect, and exploitation in NHs in NBCP states. Consistent with a previous study [24], our study suggests that NBCP is associated with fewer health-related deficiencies in nursing homes, likely due to the enhanced scrutiny and improved hiring practices resulting from comprehensive background checks. Although the number of citations related to abuse, neglect, and exploitation are fewer compared with overall health deficiencies [40], about 27% of NHs receive an abuse-related citation. We observed about 11% decline in the probability of receiving an abuse-related citation following NBCP.

To contextualize the magnitude of these findings, we estimate that the NBCP is associated with a reduction of 0.76 health deficiencies and 0.048 abuse-related citations per facility annually. Given the approximately 5200 nursing homes in the NBCP states in our sample, these coefficients can be translated to an aggregate reduction of roughly 3952 health deficiencies and 250 fewer abuse, neglect, and exploitation citations per year across the treated states. Furthermore, the 2.9 percentage point reduction in the probability of receiving an abuse citation implies that the program prevents approximately 151 NHs in NBCP states annually from having a substantiated abuse incident. These figures suggest that the NBCP has achieved both broad improvements in regulatory compliance and targeted success in preventing harm to residents.

We also find differential treatment effects of NBCP on health- and abuse-related citations across different groups potentially due to variations in program implementation across states. Across the 2010 cohort, Delaware, Connecticut, Florida, and Rhode Island met nearly all 13 NBCP requirements, while Missouri implemented partial elements of the program due to the absence of legislative authority [44]. Among 2011 cohort, West Virginia, Oklahoma, and Utah achieved most compliance with NBCP requirements, whereas Maine, California, North Carolina, and Kentucky implemented several but not all required components [45]. For states adopting in 2012 and 2013, Georgia, Minesota, Hawaii, Ohio, and Oregon met most program requirements [45, 46]. These differences in program intensity likely contributed to the heterogeneity in observed treatment effects. However, meeting NBCP requirements is just one part of the story since the data on enforcement of these requirements is unavailable. Although the specific requirements and timeline are not detailed consistently, OIG assessments provide information on some of these statespecific implementations (Table S10).

There are reasons to expect NBCP to reduce health deficiencies but heterogeneities in effect could be the result of differences in implementation or enforcement. NBCP states were more likely to adopt and develop advanced monitoring technologies, specifically the “Rap Back” systems and fingerprinting systems [47]. These systems allow for continuous monitoring of employees' criminal records after they are hired, alerting employers to new offenses. However, despite the NBCP mandate to transition to fingerprint-based checks, OIG reports indicate that not all states fully implemented these measures. Additionally, for states that implemented policies in later years, there may not have been sufficient time to observe the full effects of the NBCP.

Participation in NBCP may help improve NH quality in different ways including reductions in citations related to employment of staff with adverse actions. Participation in NBCP may result in better staffing practices that improve organizational performance and reduction in deficiencies [48, 49]. Participation in a national program such as NBCP can help nursing homes adhere to federal mandates that require the exclusion of individuals with histories of abuse from employment [50]. Non-compliance with regulations can result in deficiency citations, underscoring the importance of comprehensive background checks such as those proposed by NBCP. Similarly, participation in NBCP may also foster a culture of continuous quality improvement within nursing homes where facilities are prioritizing safe hiring practices and maintaining staffing levels, both of which may affect health deficiency citations and abuse-related citations [5, 43]. Our findings provide important evidence for policymakers debating the NBCP program. First, we observed that NBCP is associated with fewer deficiency citations across different types of nursing homes including those with higher levels of ADRD census and those located in nonmetropolitan areas. Given that residents with ADRD are less likely to report mistreatment due to their cognitive limitations [8, 14-17], the significant impact of the NBCP on reducing health deficiencies and citations for abuse, neglect, and exploitation in both settings underscores the program's importance in protecting this vulnerable population. Although residents with ADRD are more vulnerable to mistreatment, the protective mechanisms of NBCP may apply to all residents since NBCP improves staff-level background screening and hiring standards. NBCP likely improved baseline safety culture across facilities rather than generating disproportionate protection for ADRD residents. Similarly, we found that NBCP was important in reducing health deficiencies in both metropolitan and non-metropolitan NHs. There were concerns that non-metropolitan NHs may face unique challenges in staffing and may face adverse impact of NBCP but we observe meaningful reductions in health deficiencies [38, 39]. Second, we observed meaningful reductions in health deficiencies and deficiencies related to abuse, neglect, and exploitation suggesting that NBCP is effective in improving care beyond those related to abuse and neglect. Expansion of NBCP-type programs along with rigorous enforcement may enhance NH safety. Continuous support of NBCP program coupled with additional measures, such as increased training for staff and enhanced oversight, may be necessary to amplify these effects [23, 50].

Our study contributes to policy discussions on NH workforce regulation and patient safety in LTC settings by providing causal evidence on the effectiveness of staff background checks. The NBCP is not only a compliance program, but an intervention aimed at improving the integrity and competency of the NH workforce by excluding individuals with prior abuse or criminal histories from resident care. By demonstrating that NBCP participation reduces citations related to abuse, neglect, and exploitation and health deficiencies, our findings show how NH staff screening policies can enhance care safety and quality. Our results indicate the importance of regulatory mechanisms that strengthen workforce oversight and improve efforts to improve patient safety through training, monitoring, and organizational accountability in NH. Future research linking NBCP data with workforce and inspection process measures could help explore these mechanisms.

Our study is subject to several limitations. First, our study focused on citations of health deficiencies and citations for abuse, neglect, and exploitation, two of the critical indicators of nursing home quality directly related to NBCP. In future studies, it would be important to use resident-level data to evaluate resident-level quality outcomes such as falls or nursing home-level staffing and financial outcomes to get a comprehensive evaluation of NBCP. Second, deficiency citations are proxy outcomes and may be subject to measurement bias due to variation in surveyor practices, complaint volume, and state oversight cultures. Finally, states may differ in their approach to NBCP implementation, including differences in the rigor and frequency of background checks, enforcement practices, and supplementary measures. If these factors varied over time differentially across NBCP and non-NBCP states, they may potentially impact our findings. For example, we observed considerable heterogeneity in NBCP effects across groups of states that adopted NBCP in different years, suggesting that NBCP may have differential impact based on state-level policies. Future research should explore the heterogeneity in NBCP implementation using surveys of state agents responsible for background check programs. It would also be beneficial to investigate how different components of the program, such as the frequency and depth of background checks, contribute to the observed outcomes. Even with the implementation of key NBCP components across most of the states, the extent to which states are enforcing these requirements is unclear as we do not have publicly available information on this.

## 5 ∣ Conclusion

NBCP appears to be a valuable tool in improving nursing home quality by reducing health deficiencies and potentially mitigating incidents of abuse, neglect, and exploitation. These findings highlight the importance of comprehensive background checks and the need for ongoing research on policies aimed at protecting vulnerable nursing home residents.

## Supplementary Material

Suppmaterial

Additional supporting information can be found online in the Supporting Information section. **Table S1:** F-tags. **Figure S1:** Robustness check: Honest DID outputs. **Table S2:** Group treatment effects of NBCP on citations for health deficiencies, and abuse, neglect, and exploitation (Callaway and Sant'Anna Difference-in-Differences [CSDID]). **Table S3:** Group treatment effects of NBCP on citations for health deficiencies, and abuse, neglect, and exploitation (Callaway and Sant'Anna Difference-in-Differences [CSDID]) controlling full set of covariates. **Table S4:** Robustness check: Effects of NBCP on deficiencies points for health deficiencies, and abuse, neglect, and exploitation (Callaway and Sant'Anna Difference-in-Differences [CSDID]) controlling number of beds, profit status, HHI index, chain membership, CCRC status, having special care unites, and acuity scores. **Table S5:** Robustness check: Effects of NBCP on citations for health deficiencies, and abuse, neglect, and exploitation (Callaway and Sant'Anna Difference-in-Differences [CSDID]) excluding F221 and F222. **Table S6:** Robustness check: Effects of NBCP on deficiency score for health deficiencies, and abuse, neglect, and exploitation (Callaway and Sant'Anna Difference-in-Differences [CSDID]). **Table S7:** Robustness check: Effects of NBCP on citations for health deficiencies, and abuse, neglect, and exploitation (Callaway and Sant'Anna Difference-in-Differences [CSDID]) excluding hospital-affiliated NH. **Table S8:** Robustness check: Effects of NBCP on citations for health deficiencies, and abuse, neglect, and exploitation (Callaway and Sant'Anna Difference-in-Differences [CSDID]) ranging years from 2011 to 2016. **Table S9:** Placebo test: Effects of NBCP on citations for environmental citations (Callaway and Sant'Anna Difference-in-Differences [CSDID]). **Table S10:** OIG reports of NBCP implementation.

## Figures and Tables

**TABLE 1 ∣:** Descriptive statistics of nursing homes (NH) in 2009.

Variables	Full	NBCP states	Non- NBCP states
Outcome			
Number of health deficiencies	7.66	8.48	6.97
Probability of citation for abuse, neglect, and exploitation	0.27	0.29	0.25
Number of citation of abuse, neglect, and exploitation	0.35	0.39	0.32
NH characteristics			
Number of beds	108.04	99.46	115.32
Profit status			
For-profit (%)	68.42	71.46	65.84
Nonprofit (%)	25.43	23.17	27.34
Government (%)	6.15	5.37	6.82
HHI	0.23	0.21	0.24
Medicaid residents (%)	60.99	61.85	60.25
Medicare residents (%)	15.01	15.15	14.90
Private residents (%)	24.00	23.00	24.85
Occupancy rate (%)	83.39	83.82	83.02
Nurse staffing hours per resident day (HPRD)			
RNs HPRD	0.56	0.57	0.56
LPNs HPRD	0.86	0.86	0.86
CNAs HPRD	2.30	2.39	2.22
Unemployment rate (county level, %)	9.00	9.92	8.21
Crime rate per 100,000 (state level)	413.45	414.06	412.93
Metropolitan (%)	71.55	73.25	70.11
Nonmetropolitan (%)	28.45	26.75	29.89
Observations	11,333	5200	6133

*Note:* Sample observations include NBCP states awarded between 2010 and 2015 and non-NBCP states. Pilot NBCP states are excluded to avoid always-treated effect. HHI: Herfindahl–Hirschman index is calculated by market share (number of residents) at county level. HPRD is calculated by nursing staffing and administration hours and divided by number of residents. RNs are registered nurses; LPNs are licensed practical nurses; CNAs are certified nursing aides; Unemployment rate at county level is obtained from U.S. Bureau of Labor Statistics. Crime rate per 100,000 at state level is obtained from Federal Bureau of Investigation Crime Data. Metropolitan statuses are defined by 2013 Rural–Urban Continuum Codes.

**TABLE 2 ∣:** Effects of NBCP on citations for health deficiencies, and abuse, neglect, and exploitation (Callaway and Sant'Anna Difference-inDifferences [CSDID]).

Outcomes	Model 1	Model 2	Model 3
Number of health deficiencies	−0.760*** (0.094)	−0.770*** (0.097)	−0.739*** (0.094)
Full sample average: 7.66			
Probability of citation for abuse, neglect, and exploitation	−0.029*** (0.007)	−0.035*** (0.008)	−0.027*** (0.007)
Full sample average: 0.27			
Number of citation for abuse, neglect, and exploitation	−0.048*** (0.012)	−0.054*** (0.012)	−0.044*** (0.012)
Full sample average: 0.35			
*N*	88,680	88,389	90,125
Covariates	No	Yes	No
Control group	Never treated	Never treated	Never and not-yet-treated

*Note:* Treatment includes nursing homes in states with NBCP grants between 2010 and 2015 and excludes pilot NBCP states. Model 2 adjusts for covariates: number of beds, profit status, HHI index, chain membership, continuing care retirement community status, having special care units, and acuity scores.

*** *p* < 0.01.

**TABLE 3 ∣:** Effects of NBCP on citations for health deficiencies, and abuse, neglect, and exploitation (Callaway and Sant'Anna Difference-in-Differences [CSDID]) stratified by ADRD census and metropolitan status.

Outcomes	Overall	High ADRD census	Low ADRD census	Metropolitan	Nonmetropolitan
Number of health deficiencies	−0.760*** (0.094)	−0.812*** (0.127)	−0.645*** (0.142)	−0.780*** (0.113)	−0.696*** (0.170)
Full sample average: 6.908					
Probability of citation for abuse, neglect, and exploitation	−0.029*** (0.007)	−0.026*** (0.010)	−0.030*** (0.011)	−0.025*** (0.009)	−0.042*** (0.014)
Full sample average: 0.238					
Number of citation for abuse, neglect, and exploitation	−0.048*** (0.012)	−0.047*** (0.016)	−0.035** (0.017)	−0.044*** (0.016)	−0.061*** (0.022)
Full sample average: 0.357					
*N*	88,680	53.547	34,828	63,982	24,691
Control group	Never treated	Never treated	Never treated	Never treated	Never treated

*Note:* Treatment includes nursing homes in states with NBCP grants between 2010 and 2015 and excludes pilot NBCP states. Metropolitan statuses are defined by 2013 Rural–Urban Continuum Codes. Standard error clustered at the state level.

** *p* < 0.05.

*** *p* < 0.01.

## Data Availability

The data that support the findings of this study are available in CMS Care Compare site at https://www.medicare.gov/care-compare/.

## What Is Known on This Topic

- NH in the United States are home to over a million older residents, many of whom suffer from Alzheimer's Disease (AD) and Alzheimer's Disease-Related Dementias (ADRD).
- NH are regularly cited for health deficiencies including citations for abuse, neglect, and exploitation.
- NBCP was implemented to reduce abuse, neglect, and exploitation and improve quality of nursing homes.

## What This Study Found

- Overall, NBCP is effective in reducing health deficiency citations and citations related to abuse, neglect, and exploitation in US nursing homes.
- NBCP effectiveness in reducing deficiency citations differs across states.
