# Supplementary material for: Evaluating the Effect of National Background Check Program on Nursing Home Deficiency Citations
Source: Health Serv Res. Author manuscript; Available in PMC 2026 May 6. (PMC13140987; doi:10.1111/1475-6773.70108)
Supplement: Suppmaterial [file NIHMS2167254-supplement-Suppmaterial.docx]

| Appendix Table 1. F-tags | |
| --- | --- |
| Tag | Description |
| F221 | Right to be free from physical restraints |
| F222 | Right to be free from chemical restraints |
| F223 | Right to be free from abuse and neglect; Right to be free from misappropriation and exploitation; Right to be free from involuntary seclusion |
| F224 | Must not use verbal/physical/sexual abuse, corporal punishment, or involuntary seclusion |
| F225 | Not employ/engage staff with adverse actions; Report allegations of abuse, neglect, misappropriation; Investigate allegations of abuse and neglect, misappropriation and prevent further occurrence |
| F226 | Develop/implement policies prohibiting abuse/neglect/misappropriation/exploitation; Abuse, neglect, and exploitation training |

Appendix Figure 1. Robustness Check: Honest DID Outputs

Notes: Appendix Figure 1 presents estimates and 95% robust confidence intervals from Honest DID sensitivity analyses for the original estimate (M = 0) and for alternative sensitivity parameters (Mbar = 0.5, 1, 1.5, and 2).

| Appendix Table 2. Group treatment effects of NBCP on citations for health deficiencies, and abuse, neglect, and exploitation (Callaway and Sant’Anna Difference-in-Differences (CSDID)) | | | | | | |
| --- | --- | --- | --- | --- | --- | --- |
| Outcomes | ATT | Group 2010 | Group 2011 | Group 2012 | Group 2013 | Group 2015 |
|  |  |  |  |  |  |  |
| *Number of Health Deficiencies* | -0.760*** (0.094) | -1.203*** (0.167) | -0.475*** (0.140) | -1.205*** (0.238) | -0.288 (0.194) | -0.087 (0.673) |
|  |  |  |  |  |  |  |
| *Probability of Citation for Abuse, Neglect, and Exploitation* | -0.029*** (0.007) | -0.027** (0.013) | -0.023** (0.011) | -0.080*** (0.020) | -0.030* (0.017) | 0.056 (0.040) |
|  |  |  |  |  |  |  |
| *Number of Citation for Abuse, Neglect, and Exploitation* | -0.048*** (0.012) | -0.060*** (0.019) | -0.025 (0.017) | -0.165*** (0.039) | -0.038 (0.031) | 0.166* (0.085) |
| N | 88,680 | 11,324 | 18,065 | 4,900 | 7,156 | 2,126 |
| Covariates |  | No | | | | |
| Control group |  | Never treated | | | | |
| Notes: Treatment includes nursing homes in states with NBCP grants between 2010 and 2015 and excludes pilot NBCP states. Group 2010 includes Delaware, Connecticut, Florida, Missouri, and Rhode Island; Group 2011 includes California, Oklahoma, Kentucky, Utah, North Carolina, Maine, and West Virginia. Group 2012 includes Georgia, Minnesota, and Hawaii; Group 2013 includes Ohio and Oregon. Group 2015 includes Kansas. *p<0.10, **p<0.05, ***p<0.01. | | | | | | |

| Appendix Table 3. Group treatment effects of NBCP on citations for health deficiencies, and abuse, neglect, and exploitation (Callaway and Sant’Anna Difference-in-Differences (CSDID)) controlling full set of covariates | | | | | | |
| --- | --- | --- | --- | --- | --- | --- |
| Outcomes | ATT | Group 2010 | Group 2011 | Group 2012 | Group 2013 | Group 2015 |
|  |  |  |  |  |  |  |
| *Number of Health Deficiencies* | -0.062 (0.650) | -0.759 (1.275) | 0.981 (1.054) | -1.229*** (0.274) | -0.328 (0.241) | 0.062 (0.681) |
|  |  |  |  |  |  |  |
| *Probability of Citation for Abuse, Neglect, and Exploitation* | -0.100** (0.047) | -0.051 (0.127） | -0.157*** (0.046) | -0.092*** (0.024) | -0.025 (0.021) | 0.057 (0.043) |
|  |  |  |  |  |  |  |
| *Number of Citation for Abuse, Neglect, and Exploitation* | -0.072 (0.073) | 0.128 (0.194) | -0.212*** (0.071) | -0.167*** (0.045) | -0.035 (0.036) | 0.160* (0.089) |
| N | 88,680 | 11,324 | 18,065 | 4,900 | 7,156 | 2,126 |
| Covariates |  | Full | | | | |
| Control group |  | Never treated | | | | |
| Notes: Treatment includes nursing homes in states with NBCP grants between 2010 and 2015 and excludes pilot NBCP states. Full set of covariates includes payer mix (Medicaid percentage, Medicare percentage), number of beds, occupancy rate, profit status, hours per resident day, Herfindahl–Hirschman index, the unemployment rate at the county level, as well as state crime rate per 100,000 population in each state, metropolitan status). Group 2010 includes Delaware, Connecticut, Florida, Missouri, and Rhode Island; Group 2011 includes California, Oklahoma, Kentucky, Utah, North Carolina, Maine, and West Virginia. Group 2012 includes Georgia, Minnesota, and Hawaii; Group 2013 includes Ohio and Oregon. Group 2015 includes Kansas. *p<0.10, **p<0.05, ***p<0.01. | | | | | | |

| Appendix Table 4. Robustness check: Effects of NBCP on deficiencies points for health deficiencies, and abuse, neglect, and exploitation (Callaway and Sant’Anna Difference-in-Differences (CSDID)) controlling number of beds, profit status, HHI index, chain membership, CCRC status, having special care unites, and acuity scores | | | |
| --- | --- | --- | --- |
| Outcomes | Specification 1 | Specification 2 | |
|  |  |  |  |
| *Number of Health Deficiencies* | -0.727*** (0.096) | -0.798*** (0.095) |  |
| Main Analysis: -0.760 |  |  |  |
|  |  |  |  |
| *Probability of Citation for Abuse, Neglect, and Exploitation* | -0.033*** (0.007) | -0.031*** (0.007) |  |
| Main Analysis: -0.029 |  |  |  |
|  |  |  |  |
| *Number of Citation for Abuse, Neglect, and Exploitation*  Main Analysis: -0.048 | -0.052*** (0.012) | -0.050*** (0.012) |  |
| *N* | 88,680 | 88,389 |  |
| Covariates | Yes | |  |
| Control group | Never treated | |  |
| Notes: Treatment includes nursing homes in states with NBCP grants between 2010 and 2015 and excludes pilot NBCP states. Specification 1 adjusts for covariates: number of beds, profit status, and HHI index. Specification 2 adjusts for covariates: chain membership, CCRC status, having special care unites, and acuity scores. *p<0.10, **p<0.05, ***p<0.01 | | | |

| Appendix Table 5. Robustness check: Effects of NBCP on citations for health deficiencies, and abuse, neglect, and exploitation (Callaway and Sant’Anna Difference-in-Differences (CSDID)) excluding F221 and F222 | | | | |
| --- | --- | --- | --- | --- |
| Outcomes | Model 1 | Model 2 | | Model 3 |
|  |  |  |  | |
| *Number of Health Deficiencies* | -0.754*** (0.095) | -0.724*** (0.096) | -0.735*** (0.095) | |
| Main Analysis: -0.760 |  |  |  | |
|  |  |  |  | |
| *Probability of Citation for Abuse, Neglect, and Exploitation* | -0.028*** (0.007) | -0.032*** (0.007) | -0.026*** (0.007) | |
| Main Analysis: -0.029 |  |  |  | |
|  |  |  |  | |
| *Number of Citation for Abuse, Neglect, and Exploitation*  Main Analysis: -0.048 | -0.046*** (0.012) | -0.050*** (0.012) | -0.043*** (0.012) | |
| N | 87,738 | 87,738 | 89,188 | |
| Covariates | No | Yes | No | |
| Control group | Never treated | Never treated | Never and not-yet-treated | |
| Notes: Treatment includes nursing homes in states with NBCP grants between 2010 and 2015 and excludes pilot NBCP states. Model 2 adjusts for covariates: number of beds, profit status, and HHI index. *p<0.10, **p<0.05, ***p<0.01 | | | | |

| Appendix Table 6. Robustness check: Effects of NBCP on deficiency score for health deficiencies, and abuse, neglect, and exploitation (Callaway and Sant’Anna Difference-in-Differences (CSDID)) | | | | |
| --- | --- | --- | --- | --- |
| Outcomes | Model 1 | Model 2 | | Model 3 |
|  |  |  |  | |
| *Health Deficiencies* | -0.704*** (0.096) | -0.686*** (0.097) | -0.691*** (0.096) | |
|  |  |  |  | |
| *N* | 80,522 | 80,522 | 80,522 | |
|  |  |  |  | |
| *Abuse, Neglect, and Exploitation* | -0.030*** (0.007) | -0.034*** (0.007) | -0.029*** (0.007) | |
|  |  |  |  | |
| *N* | 12,930 | 12,930 | 13,427 | |
| Covariates | No | Yes | No | |
| Control group | Never treated | Never treated | Never and not-yet-treated | |
| Notes: Treatment includes nursing homes in states with NBCP grants between 2010 and 2015 and excludes pilot NBCP states. Model 2 adjusts for covariates: number of beds, profit status, and HHI index. *p<0.10, **p<0.05, ***p<0.01 | | | | |

| Appendix Table 7. Robustness check: Effects of NBCP on citations for health deficiencies, and abuse, neglect, and exploitation (Callaway and Sant’Anna Difference-in-Differences (CSDID)) excluding hospital-affiliated NH | | | | |
| --- | --- | --- | --- | --- |
| Outcomes | Model 1 | Model 2 | | Model 3 |
|  |  |  |  | |
| *Number of Health Deficiencies* | -0.719*** (0.098) | -0.690*** (0.099) | -0.701*** (0.098) | |
| Main Analysis: -0.760 |  |  |  | |
|  |  |  |  | |
| *Probability of Citation for Abuse, Neglect, and Exploitation* | -0.033*** (0.008) | -0.037*** (0.008) | -0.030*** (0.008) | |
| Main Analysis: -0.029 |  |  |  | |
|  |  |  |  | |
| *Number of Citation for Abuse, Neglect, and Exploitation*  Main Analysis: -0.048 | -0.051*** (0.012) | -0.055*** (0.012) | -0.047*** (0.012) | |
| N | 82,535 | 82,535 | 83,788 | |
| Covariates | No | Yes | No | |
| Control group | Never treated | Never treated | Never and not-yet-treated | |
| Notes: Treatment includes nursing homes in states with NBCP grants between 2010 and 2015 and excludes pilot NBCP states. Model 2 adjusts for covariates: number of beds, profit status, and HHI index. *p<0.10, **p<0.05, ***p<0.01 | | | | |

| Appendix Table 8. Robustness check: Effects of NBCP on citations for health deficiencies, and abuse, neglect, and exploitation (Callaway and Sant’Anna Difference-in-Differences (CSDID)) ranging years from 2011 to 2016 | | | | |
| --- | --- | --- | --- | --- |
| Outcomes | Model 1 | Model 2 | | Model 3 |
|  |  |  |  | |
| *Number of Health Deficiencies* | -0.683*** (0.151) | -0.686*** (0.097) | -0.691*** (0.096) | |
| Main Analysis: -0.760 |  |  |  | |
|  |  |  |  | |
| *Probability of Citation for Abuse, Neglect, and Exploitation* | -0.047*** (0.013) | -0.034*** (0.007) | -0.029*** (0.007) | |
| Main Analysis: -0.029 |  |  |  | |
|  |  |  |  | |
| *Number of Citation for Abuse, Neglect, and Exploitation*  Main Analysis: -0.048 | -0.081*** (0.024) | -0.043*** (0.012) | -0.037*** (0.012) | |
| N | 47,516 | 47,516 | 47,751 | |
| Covariates | No | Yes | No | |
| Control group | Never treated | Never treated | Never and not-yet-treated | |
| Notes: Treatment includes nursing homes in states with NBCP grants between 2011 and 2015 and excludes pilot NBCP states. Model 2 adjusts for covariates: number of beds, profit status, and HHI index. *p<0.10, **p<0.05, ***p<0.01 | | | | |

| Appendix Table 9. Placebo Test: Effects of NBCP on citations for environmental citations (Callaway and Sant’Anna Difference-in-Differences (CSDID)) | |
| --- | --- |
| Outcomes | Model 1 |
|  |  |
| *Environmental Citations* | -0.007 (0.006) |
|  |  |
|  |  |
| N | 88,680 |
| Covariates | No |
| Control group | Never treated |
| Notes: Treatment includes nursing homes in states with NBCP grants between 2010 and 2015 and excludes pilot NBCP states. *p<0.10, **p<0.05, ***p<0.01 | |

Appendix Table 10: OIG Reports of NBCP Implementation

| **OIG Report** | **Included Treatment States** |
| --- | --- |
| National Background Check Program for Long-Term-Care Providers: Assessment of State Programs Concluded Between 2013 and 2016. (OEI‑07‑16‑00160), April 25, 2019 | CT; DE; MO |
| National Background Check Program for Long-Term-Care Providers: Assessment of State Programs Concluded in 2017 and 2018. (OEI‑07‑18‑00290), August 21, 2019. | CA; GA; KY; ME; NC; OK; UT |
| National Background Check Program for Long-Term-Care Providers: Assessment of State Programs Concluded in 2019. (OEI‑07‑20‑00180), September 2020. | HI; OH; OR |
| Florida Ensured That Nursing Homes Complied with Federal Background Check Requirements. (Report No. A‑04‑23‑08100), April 29, 2024 | FL |
| Notes: Pilot states are excluded. | |
